# Clinical Proteomics: A Promise Becoming Reality

**DOI:** 10.1016/j.mcpro.2023.100688

**Published:** 2024-01-27

**Authors:** Michael A. Gillette, Connie R. Jimenez, Steven A. Carr

**Affiliations:** 1 Broad Institute of Massachusetts Institute of Technology and Harvard, Cambridge, Massachusetts, USA; 2 Massachusetts General Hospital, Boston, Massachusetts, USA; 3 Amsterdam University Medical Center, Vrije Universiteit Amsterdam, Amsterdam, Netherlands

“Clinical proteomics” can be understood to encompass a spectrum of activity from pre-clinical discovery to applied diagnostics: proteomics applied to clinically relevant materials; proteomics addressing a clinical question or need; or mass spectrometry (MS)-based or proteomics-derived tests in the reference or clinical laboratory informing clinical decision-making. Proteomics approaches are increasingly widely used to explore the biological basis of disease, identify new targetable proteins and pathways for therapeutic intervention, predict disease outcome or treatment response, and probe resistance mechanisms. This special issue of Molecular and Cellular Proteomics presents a wide range of primary research, reviews, and perspectives pertaining to clinical proteomics. The expanding scope of proteomics applications is reflected in the spectrum of disease foci represented, including neurodegenerative diseases (1, 2, 3, 4), infectious disease (5, 6), endocrinology (7, 8), cardiovascular disease (9), inflammatory disease (10), kidney disease (11), and cancer (12, 13, 14, 15), as well as in the burgeoning areas of health monitoring and exercise (16, 17).

The growth of clinical proteomics has been enabled in part by advances in instrumentation, methodologies, and computational capabilities. Methodological improvements have allowed more detailed characterization of challenging materials like the extracellular matrix (18) and detergent-insoluble CNS tissues (4). The importance of understanding extracellular vesicles (EV) and their payloads has driven development of more robust and sensitive methods for EV proteomics (19). The field of immunopeptidomics and its potential to inform biological understanding of immunological response to disease as well as to guide cancer and other vaccine development for precision medicine has expanded significantly owing to new technologies and their optimization (20). Mass spectrometry remains the workhorse of contemporary clinical proteomics, but improvements in the scale and sensitivity of array-based approaches make them an attractive alternative for select applications (1, 16). Complex cross-sectional, longitudinal and multi-omic analyses are facilitated by accelerating computer speeds, storage capabilities, and the promulgation of computational methods focused on proteomics (17, 18). Improved sample-handling methods and instrument sensitivity have contributed to the expansion of spatially-resolved proteomics applications (8). Prior technological limitations that have prevented large numbers of patient samples from being analyzed have been greatly reduced owing to highly multiplexed strategies (3, 4, 6, 8), far more sensitive MS systems, and new scanning methods such as data independent acquisition.

Targeted MS approaches such as multiple reaction monitoring have enabled development and application of quantitative assays for proteins and their modifications that are of potential clinical value for assessing the presence and stage of disease and the effects of drugs. Targeted MS assays have largely been developed for purposes of protein biomarker candidate verification in plasma but are increasingly being used in biology and preclinical studies to measure peptide and PTM-modified peptide targets in large numbers of samples. These and related MS methods such as imaging MS are being explored to augment or replace conventional tissue histopathologic approaches in the pathology suite and clinical laboratory (21, 22).

MS-based proteomics remains especially important to deepening our understanding of signaling biology and the effects of disease and drug perturbations on posttranslational modifications such as phosphorylation, ubiquitylation, acetylation, sumoylation, and glycosylation. Improved methods now enable these and many other PTMs of importance (12) to be analyzed at deep scale, individually or as part of serial enrichment and analysis strategies yielding global proteome and numerous PTMs from exactly the same sample, thereby facilitating multi-omic integration while sparing precious patient material. Such comprehensive molecular portraits are much more likely to inform our understanding of disease than genomic characterization alone. Bottlenecks such as lack of access to clinical annotation and patient follow-up information that can be integrated with the increasingly large amounts of proteomics data are slowly being reduced as the value of proteomics and proteogenomics to enhancing the understanding of disease becomes ever clearer (23).

While initially overhyped, there is now considerable evidence that biofluid-based proteomics using MS and other techniques has the depth, sensitivity and reproducibility required to identify promising candidate biomarkers to aid in diagnosis, prognosis, and clinically-actionable prediction (1, 2, 24, 25, 26). The needs for additional validation, assay refinement, health economics assessment, and viable reimbursement strategies remain challenges to the deployment of such biomarkers in the clinic.

The application of proteomics to clinical problems and its translation to the clinic remain works in progress, but as the papers in this issue demonstrate, the potential is greater than ever, as is the likelihood of having direct, positive impact on patients through identification of new targets and therapeutic interventions and the more precise use of existing therapies.

## Conflict of interest

S. A. C. is a member of the scientific advisory boards of Kymera, PTM BioLabs, and Seer. M. A. G and S. A. C. are members of the scientific advisory board of PrognomIQ.
